# Initial Experience with Fenestrated Physician-Modified Stent Grafts Using 3D Aortic Templates

**DOI:** 10.3390/jcm11082180

**Published:** 2022-04-13

**Authors:** Paweł Rynio, Tomasz Jedrzejczak, Anita Rybicka, Ross Milner, Piotr Gutowski, Arkadiusz Kazimierczak

**Affiliations:** 1 Department of Vascular Surgery, Pomeranian Medical University in Szczecin, 70-111 Szczecin, Poland; piotr_gutowski@poczta.onet.pl (P.G.); biker2000@wp.pl (A.K.); 2 Department of Cardiac Surgery, Pomeranian Medical University in Szczecin, 70-111 Szczecin, Poland; tom.jedrzejczak@gmail.com; 3 Department of Nursing and Health Sciences, Pomeranian Medical University, 71-210 Szczecin, Poland; anitarybicka@onet.eu; 4 Center for Aortic Diseases, Chicago, IL 60637, USA; rmilner@surgery.bsd.uchicago.edu

**Keywords:** fenestrated endovascular repair, physician-modified stent graft, 3D printing, 3D model, 3D template, complex aortic procedure

## Abstract

The goal of this study was to describe the surgical results of physician-modified endografts (PMEG) utilizing a 3D aortic template in a center with no prior experience in complex endovascular aortic repairs. Forty-three patients underwent physician-modified graft stent implantation using a 3D aortic model. The inclusion criteria were juxtarenal and suprarenal aortic aneurysms, type IV thoracoabdominal aneurysms, and type IA endoleak after endovascular aortic repair. In asymptomatic patients, the diameter threshold for aneurysm repair was 5.5 cm in males and 5.0 cm in females. 3D aortic templates were prepared from the patient’s computed tomography angiography scans and sterilized before use in the operating suite. Forty-three stent grafts were modified with the use of a 3D printing template. A total of 162 reinforced fenestrations (37 celiac, 43 right renal, 39 left renal, 43 superior mesenteric) with a mean of 3.8 per patient were performed. All PMEGs had a posterior reducing-diameter tie and a preloaded guidewire. The mean modification time was 86 ± 12 min. The mean follow-up was 14 ± 12 months. The 30-day mortality was 12%. During the follow-up period, the patency rate was 95% per the superior mesenteric artery, 93% per right renal artery, 95% per left renal artery, and 89% per celiac trunk. Twelve (28%) patients had endoleak, of which type I or III was present in 5 (12%) patients, and type II in 7 (16%). 3D printing can be successfully integrated into the physician’s everyday practice of stent graft modification. However, the use of this approach in centers without experience performing complex aortic procedures results in worse surgical metrics than those previously reported.

## 1. Introduction

Fenestrated endovascular aortic aneurysm repair (FEVAR) and branched endovascular aortic aneurysm repair (BEVAR) have become the first-choice therapies for pararenal and thoracoabdominal aneurysms, especially in patients unfit for open surgery [1]. Fenestrations and branches enable the maintenance of aortic side branches and, as a result, more proximal sealing zone extensions. However, these stent-grafts are not commonly available as off-the-shelf products and have anatomy-related constraints. Waiting times for custom-made devices (CMD) can be as long as 12–15 weeks, depending on your geographic location. This waiting period is associated with a cumulative mortality rate of 4% [2,3]. As a result, CMD cannot be employed in urgent cases or pathologies carrying a significant risk over time.

Alternatively, the physician can modify the stent graft (PMEG) [4]. On computed tomography angiography (CTA), the locations of the aortic side branches are identified, and these data are subsequently used in the operating room to designate the position of the fenestrations [5]. This method, however, is vulnerable to inter-observer variability [6]. Furthermore, different modification methods are applied in various centers, resulting in a lack of standardization [7,8]. The absence of quality control of the PMEG is also a criticism of the approach [7,8].

In recent years, 3D printing has enhanced PMEG to address these limitations of the technique [9]. After a modeling stage, CTA images are converted into 3D models of the aorta, which are then fabricated in a 3D printer. The 3D aortic templates are exact representations of the vascular system and exhibit exceptional accuracy compared to the source CTA scans [10]. The stent graft is thought to fit within the aortic template and thus simulates its aortic wall apposition in vivo, which might lead to an improved fenestration arrangement relative to the target vessel. Furthermore, the 3D aortic template allows for identifying a common location for multiple fenestrations where no stent strut will pass through. Because of these advantages, 3D printing is now being used to aid stent graft modifications [4,11,12].

The purpose of this study was to report the surgical results of PMEG using a 3D template in a center with no previous experience in complex endovascular aortic repairs.

## 2. Materials and Methods

From 2018 to 2021, 43 patients were eligible for FEVAR with stent graft modifications using a 3D aortic template. The inclusion criteria were juxtarenal and suprarenal aortic aneurysms, type IV thoracoabdominal aneurysms, and type IA endoleak after endovascular aortic repair. In asymptomatic patients, the diameter threshold for aneurysm repair was 5.5 cm in males and 5.0 cm in women. At aortic team meetings, all cases were discussed and rated as high risk for open surgery. The local bioethical committee authorized the study, and all patients gave their informed consent.

### 2.1. 3D Aortic Template Creation

A vascular surgeon performed the aneurysm segmentation using 3D Slicer 11.0 software (version 4.11.0, https://www.slicer.org/, accessed on 1 February 2022) [13]. All of the side vessels were cut off. The model’s wall thickness was set at 1.5 mm. The data was then uploaded to PreForm software (Formlabs, Somerville, MA, USA), which planned the 3D model’s location in the printer’s working chamber, as well as the positioning of supports. A Form 2 printer (Formlabs, Somerville, MA, USA) was used for rapid prototyping. Rinsing with isopropyl alcohol, drying, curing, and removing supports were all part of the postprocessing procedure. Models were sterilized with hydrogen peroxide plasma or ethylene oxide gas before being used in the operation room [14].

### 2.2. Stent Graft Modification

**Figure 1:**
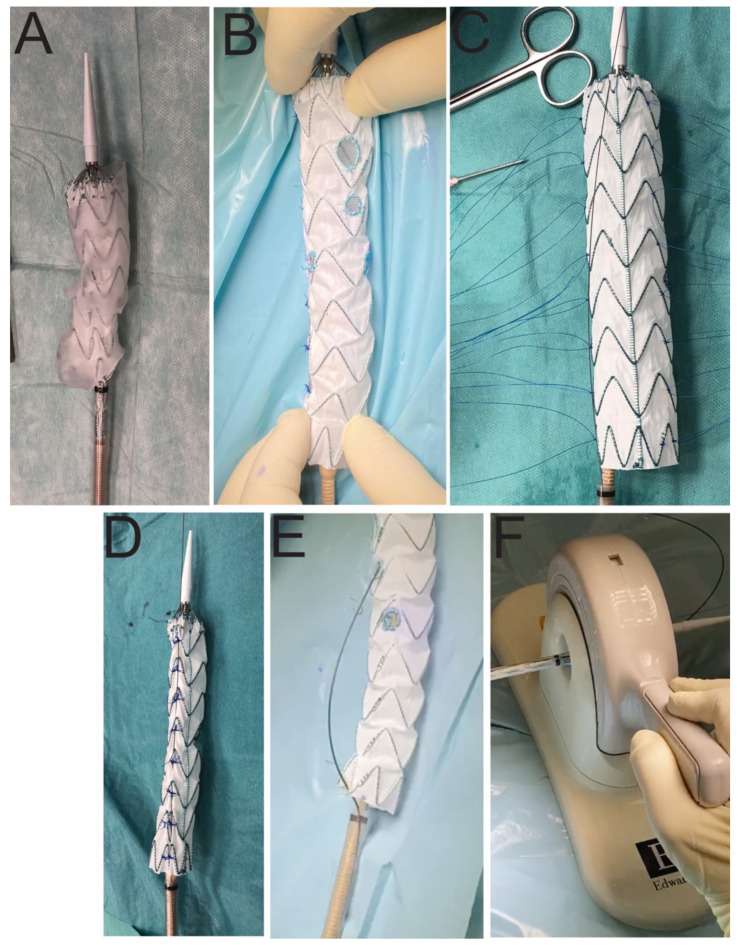
Stages of stent graft modification. Stent graft deployed in the 3D aortic template (**A**). Stent graft with created fenestrations (**B**). A step of reducing-tie creation (**C**). Reduced-diameter stent graft (**D**). A preloaded guidewire to the superior mesenteric artery (**E**). Stent graft resheathing using the aortic valve crimper (**F**).

All stent graft modifications were performed on a back table in the operating room during anesthesia and vascular access preparation (Figure 1). The platform for the modification was the Valiant Captiva (Medtronic, Dublin, Ireland). Following the 3D aortic template application, the positions of the target vessels were indicated (Figure 1). It was crucial to position the 3D template to avoid interfering with the stent struts. We intended to implant the stent graft in the low supra-celiac zone, so we prepared fenestrations for both renal arteries, the superior mesenteric artery (SMA), and the celiac trunk (CT) in every case where they were patent. A snare loop wire (Indy OTW Vascular Retriever, Cook Medical, Bloomington, IN, USA) and an embolization loop (Nester, Cook Medical, Bloomington, IN, USA) that served as radiopaque markers were used to strengthen the fenestrations (Figure 1). A posterior tie composed of a V-18 ControlWire Guidewire (Boston Scientific, Marlborough, MA, USA) was used to reduce the size of the stent-graft (Figure 1). The insertion system was perforated, and a soft guidewire was introduced via the SMA fenestration (Figure 1). This preloaded guidewire served as rapid cannulation from the axillary artery as SMA is perceived as the most important vessel. Then, the stent-graft was packed into the insertion system using an aortic valve crimper (Figure 1).

### 2.3. Endovascular Procedure

**Figure 2:**
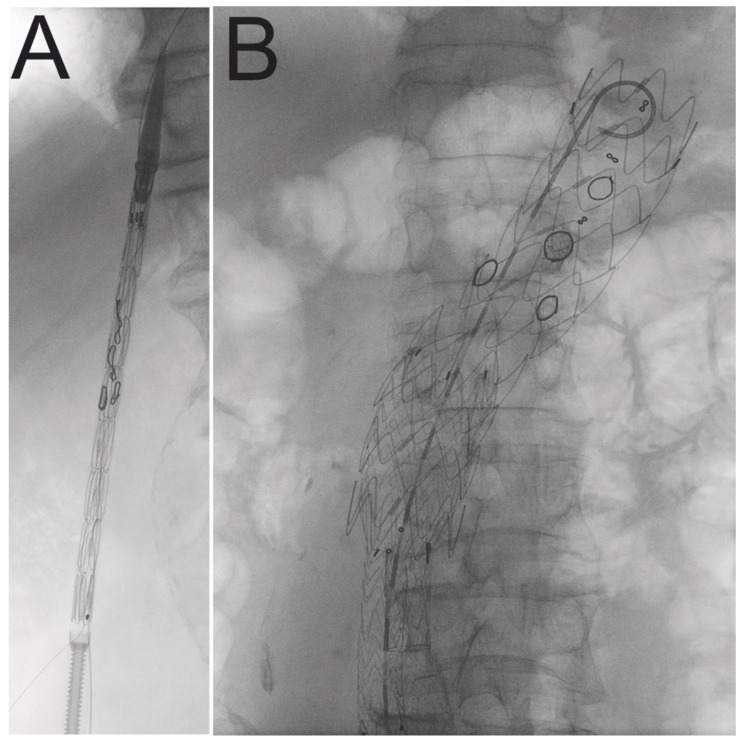
Fluoroscopy view of modified stent graft including radiopaque fenestrations markers. Stent graft before (**A**) and after (**B**) deployment.

Both common femoral arteries were exposed or treated percutaneously with the Proglide device (Abbott, Chicago, IL, USA), depending on the vascular calcification state. This approach was used for PMEG advancement under Ziehm imaging guidance (Siemens, Munich, Germany) (Figure 2). In all cases PMEG was proximally deployed in zone 5 (above the celiac trunk). A snared-out preloaded soft guidewire was utilized to cannulate the SMA through the axillary artery. The left renal artery (LRA), the right renal artery (RRA), and SMA were targeted with balloon-expandable stents, while CT was left unstented. The bridging stents were from various companies: LifeStream (BD, Franklin Lakes, NJ, USA), Advanta V12 (Getinge, Goteborg, Sweden), BeGraft (Bentley InnoMed GmbH, Hechingen, Germany). A 10 mm × 20 mm angioplasty balloon was used to flare the stents. The distal stent graft component, which includes Endurant IIs (Medtronic, Dublin, Ireland), AFX 2 (Endologix, Irvine, CA, USA), Valiant Captiva extension (Medtronic, Dublin, Ireland), and Excluder (Gore, Newark, NJ, USA), was employed whenever necessary. For asymptomatic patients who had an aortoiliac stent graft implanted, the complete exclusion of the aneurysm from the circulation was split into two stages. The contralateral limb was implanted two to four weeks following the first stage. The staged procedure was designed to reduce the risk of paraplegia by temporary aneurysm sac perfusion [15]. Additionally, to reduce the risk of paraplegia, a hemoglobin level greater than 7 mmol/L and the mean arterial pressure greater than 90 mmHg were maintained. Drainage of cerebrospinal fluid was not routinely used. After the procedure, all patients were advised dual antiplatelet therapy. All patients were compliant with medical treatment.

### 2.4. Statistical Analysis

Statistica 13.3 (TIBCO Software, Palo Alto, CA, USA) was used to conduct the statistical analysis. The mean, standard deviation, median, and interquartile range were used to describe continuous variables. Absolute numbers and percentages were used to represent categorical variables. On Kaplan–Meier estimators, target vessel patency, survival, the existence of endoleaks, and reinterventions were presented. The research was halted when an interval had fewer than ten patients at risk. Nominal variables were compared using the exact Fisher test when performing subgroup analysis. The survival comparison was performed using the logrank test. A *p*-value less than 0.05 was considered statistically significant. The aneurysm type, endoleaks, and technical success were defined using reporting standards [16].

## 3. Results

### 3.1. Patients’ Characteristics

There were 43 patients, 37 (86%) male and 6 female (14%), with a mean age of 74 ± 7 years (Table 1). Aneurysm classification was juxtarenal abdominal aortic aneurysm (AAA) in 17 (40%), suprarenal AAA in 5 (12%), type 1A endoleak in 11 (26%), visceral penetrating aortic ulcer (PAU) in 3 (7%), anastomotic aneurysm in 4 (9%), and type IV thoracoabdominal in 3 (7%). The mean aneurysm diameter was 62 ± 6 mm. Hypertension (95%) was the most prevalent comorbidity, followed by coronary artery disease (44%) and chronic renal disease (44%).

### 3.2. 3D Template Creation

All 3D aortic templates were successfully printed and sterilized. The mean 3D-printing time was 361 ± 114 min, which required 24 ± 10 mL of standard clear resin. The mean model cost was calculated based on the consumable market price and was 5 ± 2 USD.

### 3.3. A Physician Modified Stent Graft Design

Forty-three stent grafts were modified with the use of a 3D printing template. A total of 162 reinforced fenestrations (37 celiac, 43 right renal, 39 left renal, 43 superior mesenteric) with a mean of 3.8 per patient were created. All PMEGs had a posterior reducing diameter tie and a preloaded SMA guidewire. The mean modification time was 86 ± 12 min.

### 3.4. Endovascular Repair

We operated on 25 (58%) patients in one stage, and 18 (42%) in two stages. The mean operation time was 247 ± 70 min. The distal stent graft component was used in 29 cases, including three AFX, fourteen Excluder, nine Endurant IIs, and three Valiant Captiva thoracic extensions. The mean used contrast volume was 218 ± 37 mL. Technical success, defined as successful PMEG implantation and target vessel incorporation without death within 24 h from surgery, was achieved in 86% of patients. During the first postoperative day, we lost two patients. One died from complications after an iliac artery rupture, and the other from an arrhythmia. Two technical failures were related to damage to the bridging stent (superior mesenteric and renal artery) during insertion of the iliac limb extension, which led to its crushing. On two occasions, we were unable to cannulate the renal artery. In total, we implanted 124 bridging stents out of 126 planned.

### 3.5. Early Clinical Outcomes

There were five (12%) deaths during the first 30 days post-surgery. In addition to the two mentioned above, the other three resulted from multisystem organ failure. One additional death during hospital stay was due to pulmonitis and paraplegia (in total, one case—2%), constituting 14% of in-hospital mortality. The other major adverse events were acute kidney injury in three (7%) patients (none required permanent dialysis) and stroke in one (2%). We had no cases of myocardial infarction. Eight (18%) patients required ICU stays, while the mean hospital stay was 8 ± 12 days. During the second stage, patients remained at the hospital for a mean of 4 ± 2 days.

### 3.6. Late Clinical Outcomes

**Figure 3:**
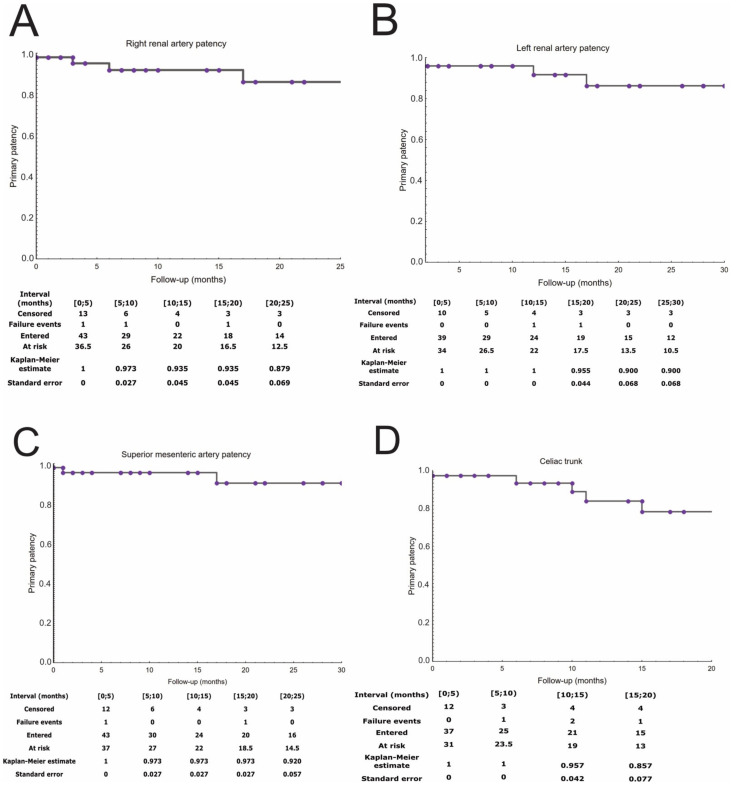
The target vessel patency rates are presented on Kaplan–Meier curves. Right renal patency (**A**). Left renal artery patency (**B**). Superior mesenteric patency (**C**). Celiac trunk patency (**D**).

**Figure 4:**
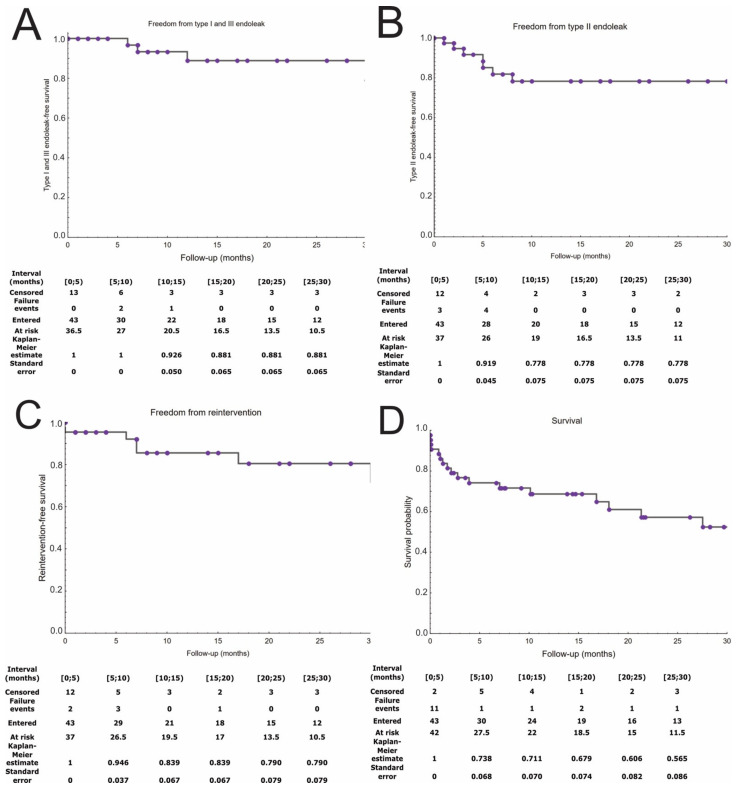
Clinical outcomes presented on Kaplan–Meier curves. Type I and III endoleak-free survival (**A**). Type II endoleak-free survival (**B**). Reintervention-free survival (**C**). Survival (**D**).

The survival, target vessel patency, reinterventions, and endoleak occurrence were presented in Kaplan–Meier estimates (Figure 3 and Figure 4). The mean follow-up was 14 ± 12 months. During the follow-up period, the patency rate was 95% per SMA, 93% per RRA, 95% per LRA, and 89% per CT. One RRA occlusion was due to cannulation failure during the index procedure. Twelve (28%) patients had endoleak, of which type I or III was present in five (12%) patients and type II in seven (16%). There were two cases of patients with Ic endoleaks, two with IIIc endoleak, one with Ib, and one with an undefined Ic/IIIc endoleak. All patients with high-pressure endoleaks underwent reintervention, as well as one patient with an endoleak from the inferior mesenteric artery. In addition, a patient who presented with acute SMA and both renal stents thrombosis was reoperated upon. Two early reinterventions were performed due to rupture of the iliac artery and closure of the SMA stent. Overall, this resulted in a reintervention rate of 19%. There were overall 17 deaths (40%) during the follow-up period. Beyond the short-term observation, one death was procedure-related in a person with acute three bridging stents thrombosis, which presented 540 days after the initial surgery. The others were gastrointestinal bleeding, general surgery complications, acute pancreatitis, and pulmonitis in one patient each, neoplasm in two, and unknown in four. Overall procedure related mortality was 16.27%.

### 3.7. Subgroup Analysis

**Figure 5:**
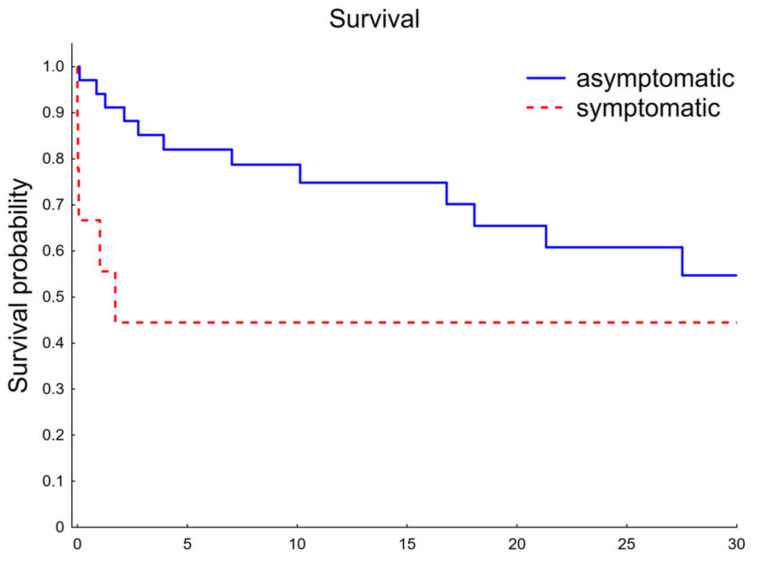
Survival comparison between asymptomatic and symptomatic subgroup.

There were nine symptomatic and thirty-four asymptomatic patients. The 30-day mortality was 5.88% in the asymptomatic subgroup and 33.33% in the symptomatic subgroup (*p* = 0.0533). In-hospital mortality was higher for symptomatic patients (33.33% vs. 8.82%); however, the difference was not statistically significant (*p* = 0.0948). There was no difference regarding technical success (asymptomatic 88.23% vs. symptomatic 77.77%; *p* = 0.5892). The log-rank test was not statistically significant for renal arteries and mesenteric arteries patency comparison. Similar, not statistically significant results were obtained for freedom from type I and III endoleak and freedom from type II endoleak. The log-rank test was statistically significant (*p* = 0.0242) for survival comparison (Figure 5). However, it should be underlined that the small size subgroup constitutes an important limitation for data interpretation.

## 4. Discussion

Here, we present a cohort of patients treated by implantation of a fenestrated physician-modified stent graft using a 3D aortic template. The outcomes presented here reflect the early stages of a single center’s learning curve for 3D printing, stent graft modification, and complex aortic procedures. Prior to this study, no FEVAR or BEVAR operations were performed at our institution. Thus, the work reflects the outcomes that other centers might anticipate when starting similar programs.

Initiation of a fenestrated stent-graft implantation program is associated with the development of new competencies among vascular surgeons, anesthesiologists, and nurses. Aortic templates are constructed using CT images of the aneurysm that have been segmented; the outer wall is then modeled and transferred to a format compatible with a 3D printer. While the 3D aortic templates are an accurate representation of the CT data, their production requires sufficient knowledge. Involvement of an experienced individual in CTA scans interpretation is a significant convenience that results in a shortened training period. A potential inaccuracy in determining the extent of aneurysm segmentation, and therefore the 3D template, could result in an improper fenestration design, and so the eclipse phenomenon during implantation. The process of fabricating the aortic template must be integrated into the hospital’s internal procedures. Modification of stent grafts is another important skill. To do this, the surgeon must be proficient with the release and resheathing of the stent graft, as well as creating the preloaded wire and creating the stent graft reducing-diameter tie. Once the right equipment is selected, it is prudent to practice on demo or overdue stent grafts outside the operating room. Additionally, endovascular skills, such as the accurate release of a fenestrated stent graft, expansion of covered stents, and flaring, are necessary. The ability to rotate and advance the stent graft can help facilitate the cannulation of mesenteric or renal arteries. Bail-out skills can be beneficial in a variety of anatomical settings. Anesthesiologists are responsible for intraoperative and perioperative patient management, and they must adapt their current practices to successfully prevent paraplegia events. Additionally, the logistics procedure is critical. Catheters, stents, guidewires, and vascular shirts must all be readily available.

Physician-modified stent grafts are readily used in settings with limited access to CMDs. These limitations may be due to inadequate funding of health care systems or the unavailability of these devices in some countries. Another important indication for the use of PMEGs are patients with symptomatic or giant aneurysms in whom waiting for a custom stent graft to be manufactured carries an unacceptably high risk of rupture. Stent grafts of this type are also employed in circumstances where CMD construction is contraindicated due to unusual anatomy. Due to these restrictions on access to CMDs, surgeons may lack experience performing complex aortic surgeries. In this case, the acquired experience with these operations will occur during the PMEG implantation.

Georgiadis and colleagues conducted a systematic review of PMEGs [17]. The 30-day mortality rate was 3.2% (1.3 to 7.2%). At short- and medium-term follow-up, total mortality was 10.7% (6.8 to 16.3%), aneurysm-related mortality was 1.1% (0.2 to 4.3%), type I endoleak rate was 1.1% (0.2 to 4.3), and type III endoleak rate was 1.6% (0.4 to 7.0%). Similarly, good results were obtained for PMEG using a 3D template [12,18]. Branzan et al. presented no deaths during a 30-day follow-up, a technical success rate of 100%, a mean ICU stay of 2.8 days, a hospital stay of 17.3 days, 5.3% branch occlusion, and 10.6% reintervention during an 11.3-month follow-up of 21 patients [12]. Tong et al. presented 3% early mortality, 3% stroke, and 14.7% leakage during a mean follow-up of 8.5 months of 34 patients treated primarily for aortic dissection [18]. Some data suggest that PMEGs may have comparable durability and mid-term clinical results as CMD [19].

Several authors have described the effect of the learning curve on procedural metrics [20,21,22]. Mirza et al. showed significant differences in surgical outcomes between the first (*n* = 81) and fourth quartile (*n* = 85) patients treated by FEVAR or BEVAR. Early mortality decreased from 6% to 0% throughout the study, while the technical difficulty of procedures increased through a higher number of incorporated target vessels from an average of 2.8 to 3.7 per patient. Major adverse events and target vessel instability decreased. Mirza et al. suggest that event rates decrease every 32 cases treated. At the same time, other authors demonstrated a steadily decreasing complication rate [22]. Concerning these data, our learning curve may be steeper due to starting with the implantation of four fenestrated stent grafts.

Because this was our initial experience with this technique, we changed the method for determining fenestration position during the study. Initially, we expanded the stent graft in a 3D aortic model and marked the target vessels’ ostia. We then performed a posterior tie, which led to a reduction in the diameter of the stent graft, but also a shift of the renal artery fenestration toward 6 o’clock on the clock face. Since the 32nd case, the method has evolved, resulting in a change in the order of stent graft modifications. The described change in technique occurred as a result of treating a patient with a ruptured aneurysm (not included in this series), where the key was to reduce the time to prepare the modified stent graft. Fenestrations were marked on the diameter-reduced stent graft, leading to a better orientation to the target vessel. When fenestrations are performed on a fully expanded stent graft and subsequently the diameter of the stent graft is reduced by putting a diameter-reducing tie, the renal artery fenestrations shift posteriorly. This shift makes cannulation of the target vessel more challenging. For this reason, it is preferable to create fenestrations on an already diameter-reduced endograft, which results in coaxial fenestrations’ alignment with the target vessels. Both unsuccessful renal artery cannulation attempts occurred prior to changing the technique. The conceptual shift demanded a redesign of the 3D aortic template, which was devoid of its posterior wall perimeter to accommodate the placement of a reduced-diameter stent graft.

Two situations involving damage to the deployed bridging stent during the advancement of the distal components of the infrarenal stent graft occurred during the study. This happens when the guidewire runs directly adjacent to the fenestration, and then advancing the graft stent insertion system afterward causes collision and damage to the covered stent. To prevent these events, we have changed the surgical technique. It is mandatory to implant the fenestrated and infrarenal stent graft modules during one procedure. The maximum possible number of bridging stents should be deployed after the infrarenal stent graft module is implanted. For this reason, our preference has become to cannulate two fenestrations from the axillary artery access. These stents should be expanded after the implantation of the infrarenal module. A covered stent to the renal artery cannulated from the contralateral femoral artery is critical. It is deployed before the deployment of the infrarenal stent graft module. Therefore, the position of a guidewire must be carefully traced in at least two fluoroscopy projections to confirm the lack of collision.

Some of the technical errors are due to the development of new technology and are difficult to avoid in their infancy. Others, such as those involving surgical techniques, can be mitigated by experience transfer. Proctoring is used routinely in complex aortic cases treated with CMD. As such, it is appropriate to gain proficiency in FEVAR and BEVAR procedures before launching stent graft modification programs utilizing a 3D aortic template. However, it must be emphasized that regional economic and political conditions of MedTech companies may preclude the use of CMD and take advantage of the proctoring service.

## 5. Conclusions

In summary, 3D printing can be successfully integrated into the physician’s everyday practice of stent graft modification. However, the use of this approach in centers without experience performing complex aortic procedures results in worse surgical metrics than those previously reported. In such conditions, it could be considered in symptomatic cases where a higher mortality rate may be acceptable.

## Figures and Tables

**Table 1:** Clinical and demographic patient characteristics and clinical outcomes.

Variable	N = 43	±SD or %	Median (Q1, Q3)
Age	73.84	±6.99	74 (68, 79.5)
Age > 80	9	20.93%	
Male gender	37	86.05%	
Hypertension	41	95.35%	
Coronary artery disease	19	44.19%	
Coronary artery bypass grafting	7	16.28%	
Arrhythmia	9	20.93%	
Heart failure	5	11.63%	
Diabetes mellitus	8	18.60%	
Chronic kidney disease	19	44.19%	
Stroke	3	6.98%	
Peripheral artery disease	8	18.60%	
Post endovascular aortic repair	11	25.58%	
GFR pretreatment	63.81	±19.92	67 (52.5, 79)
Aneurysm diameter	61.67	±6.3	62 (55, 66.5)
Aneurysm type			
Juxtarenal	17	39.53%	
Suprarenal	5	11.63%	
Type IV thoracoabdominal	3	6.98%	
Penetrating aortic ulcer	3	6.98%	
Anastomotic	4	9.30%	
Type 1A endoleak	11	25.58%	
Symptomatic	9	20.93%	
Asymptomatic	34	79.07%	
3D printing metrics			
Time	361.2	±114.09	346 (284.5, 413)
Resin volume (mL)	24.14	±10.11	22 (18, 28)
Model cost (USD)	4.99	2.09	4.55 (3.72, 5.79)
Stent graft modification metrics			
Number of fenestrations			
1	0	0.00%	
2	1	2.33%	
3	8	18.60%	
4	34	79.07%	
Average fenestrations per patient	3.77		
Target vessel fenestration			
Right renal artery	43	100.00%	
Left renal artery	39	90.70%	
Superior mesenteric artery	43	100.00%	
Celiac trunk	37	86.05%	
Operative metrics			
Percutaneous femoral access	16	37.21%	
Open femoral access	27	62.79%	
Number of bridging stents			
Planned	126		
Implanted	124		
Intended free fenestration	36		
Distal landing component			
AFX 2	3	10.35%	
Endurant IIS	9	31.03%	
Excluder	14	48.28%	
Valiant Captiva extension	3	10.35%	
Two-stages treatment	18	41.86%	
One-stage treatment	25	58.14%	
Total contrast material volume (mL)	217.67	±36.70	220 (220, 220)
Stent graft modification time	85.9	±11.52	86 (78, 94.5)
Total operating time	247.07	±69.71	250 (202.5, 280)
Technical success	37	86.05%	
Early clinical outcomes			
Paraplegia	1	2.33%	
Stroke	1	2.33%	
Myocardial infarction	0	0.00%	
Acute kidney injury	3	6.98%	
ICU stay	8	18.60%	
Hospital stay (days)	8.06	±12.49	6 (4, 8)
30-day mortality	5	11.63%	
In-hospital mortality	6	13.95%	

## Data Availability

The data that support the findings of this study are available from the corresponding author upon reasonable request.
